# Evaluation of Per- and Polyfluoroalkyl Substance (PFAS) Levels in Drinking Water: A Study of Riverside Municipal Wells

**DOI:** 10.7759/cureus.85995

**Published:** 2025-06-14

**Authors:** Shanmukh Bachhu, Quang La, Nehal Revuri, Aiman Baloch, Francis Pryor, Noman Sadiq

**Affiliations:** 1 Civil Engineering, University of California Berkeley, College Station, USA; 2 Civil Engineering, The Innovative STEMagazine 501(c)3, College Station, USA; 3 Medicine, The Innovative STEMagazine 501(c)3, College Station, USA; 4 Neurological Surgery, The Innovative STEMagazine 501(c)3, College Station, USA; 5 Medicine, Mekran Medical College, Turbat, PAK; 6 Medicine, Lake Erie College of Osteopathic Medicine, Erie, USA; 7 Physiology, Mekran Medical College, Turbat, PAK

**Keywords:** aqueous film-forming foam (afff), drinking water contamination, granular-activated carbon, groundwater pollution, ion-exchange resins, polyfluoroalkyl substances (pfas), reverse osmosis membranes, riverside municipal wells

## Abstract

Polyfluoroalkyl substances (PFAS) seem to be in every aspect of our modern-day life. PFAS chemicals manufactured by DuPont and 3M are found in our food wrappers, pots, pans, firefighting foam, and anything nonstick. The abundance of these chemicals raises concerns about the contamination of our drinking water sources. Since these chemicals get washed up by rainwater runoff, they ultimately end up in our groundwater sources. Once in our groundwater, the PFAS-contaminated water gets pumped up by wells utilized for our drinking water. In this project, our team looked at the contamination of these chemicals in our local community. Our team collaborated with local officials to gain access to sample the Garner C well, the Twin Springs well, a local private well in our community, and the Palmyrita treatment plant, where we measured both the influent and effluent water. Our team then conducted tests on each water sample to determine the amount of perfluorooctanoic acid (PFOA), perfluorooctane sulfonate (PFOS), perfluorobutanoic acid (PFBA), perfluorohexane sulfonate (PFHxS), perfluorononanoic acid (PFNA), perfluoroheptanoic acid (PFHpA), and perfluorodecanoic acid (PFDA) in each of our samples using Environmental Protection Agency (EPA) 533 rules and procedures. Using the tests from the Palmyrita treatment plant, we determined how effective Riverside’s treatment process is for removing PFAs across its water. This paper is intended to inform individuals how to test and sample for PFAS from their local wells and educate readers on PFAS contamination in the Riverside Municipal area. This study aims to quantify PFAS levels in Riverside Wells and evaluate the effectiveness of local water treatment processes, especially activated carbon filtration.

## Introduction

Polyfluoroalkyl substances (PFAS) are substances that had their start in nonstick cookware in the mid-1900s and were sold by a company known as DuPont [1]. These substances slowly grew in popularity, especially in consumer products, because they were able to repel oil, water, and grease [2]. Eventually, industries in the military, first responders, and construction were also attracted to the use of PFAS as it could extinguish fires and prevent re-ignition [3]. PFAS, however, was found to be quite toxic to the human body, as human exposure to PFAS led to altered metabolism, fertility, fetal growth, increased risk of cancers, reduced ability of the immune system to fight infections, and increased cholesterol [4]. The chemistry behind PFAS is that these substances are chains of carbon atoms that have bonded to fluorine atoms, which is what allows for their unique property of heat resistance [5]. This particular bond, as well, is one of the strongest bonds in nature, giving it the name of “forever chemicals,” as it does not degrade easily [6]. In addition, PFAS chemicals have extremely low intermolecular forces (IMF), which make them extremely hydrophobic [7].

These hydrophobic properties make PFAS extremely useful for nonstick cookware, food packaging paper, paints, stain-repellent and waterproof clothing, cosmetics, and fireproof foams [8]. They have also been reported to be in our water supply, firefighting foam, and other industrial products [9]. The widespread use of PFAS in industrial processes such as fluoropolymer manufacturing, consumer products, and firefighting foam has resulted in these chemicals being released into the environment, where they impact our drinking water through underground aquifers. People unknowingly consume this water, affecting their body function and causing people permanent damage [10].

Once PFAS gets on the ground through landfills, waste streams, industrial sites, wastewater treatment plants, and fire-fighting foam, they end up in groundwater sources due to rain [7]. PFAS can also enter a person’s body through cooking because of the abundance of PFAS in common cooking appliances such as a simple non-stick pan [11]. When someone uses these pans, PFAS gets into the food, and when consumed, the PFAS enters the bloodstream, urine, and poop. After expelling the waste, the PFAS enters the water cycle [4]. The US Environmental Protection Agency (EPA) has currently set the limit of PFAS in drinking water to be 4 ppt (parts per trillion) for perfluorooctanoic acid (PFOA) and perfluorooctane sulfonate (PFOS), where they also banned all production of PFAS in the United States of America [9]. Although the production of PFAS is banned in America, it is still being brought in from other countries, polluting our ecosystem even more. Due to California’s dry climate, most of the well contamination is linked to firefighting foam [9]. Aqueous film-forming foam (AFFF) is the main culprit in this, as AFFF contains a high amount of PFAS, causing our water and ecosystem to be polluted. PFOS is the main type of PFAS in AFFF, whereas PFOA is also formed in the manufacturing process of AFFF [7]. Thus, during our testing, we must check for PFOA and PFOS to see if our various well waters are under the 4 ppt limit. If the effluent water for the treatment plant is over 4 ppt, this would mean Riverside water would be unsafe for human consumption, and if the groundwater levels had a number greater than 4 ppt or 4 ng/L, the water would be unsafe for the ecosystem.

There are many problems for residential health when it comes to PFAS contamination. Exposure to and absorption of high levels of PFAS chemicals can result in many side effects and harmful symptoms that can be damaging to one’s health. Studies show that PFAS may negatively affect vaccine response in children and cause small decreases in infant weight. Recently, research conducted in the Cape Fear region of North Carolina has shown that the PFAS levels in their drinking water caused many disastrous cases for its younger residents [12]. Many of the infants have been born with nervous system damage, cardiac defects, and, in some extreme cases, death [12]. PFAS also causes increased cholesterol levels, changes in liver enzymes, and an increased risk of high blood pressure [13]. Animal studies have shown that PFAS damages the liver and other immune system responses and causes cancer in the kidneys and testicles [14]. According to Fenton et al., PFAS chemicals have altered thyroid function and lipid and insulin dysregulation [10]. These effects cause long-term damage to local towns and children. Although it is possible to get PFAS out of our water, it is not possible to get PFAS out of our bodies; the reality of the situation shows how PFAS is truly a "forever chemical" [10].

PFAS can cause large environmental damage and damage to many organisms at once. Although America’s water sources are already contaminated with PFAS, there are possible solutions to help with this issue. Studies have shown that certain microbial fungi, such as white rot fungus, show promise as a solution to remediate PFAS, although white rot fungus cannot be nurtured in a lab easily [15]. Ion-exchange resins act as tiny, powerful magnets in removing PFAS in our water because they are made up of hydrocarbons. There are two types of ion exchange resins, anionic and cationic, where negatively charged cationic exchange resins (CER) are effective in removing positively charged contaminants. Anionic exchange resins (AER) are effective in removing negatively charged contaminants such as PFAS. Although ion exchange resins are more expensive than other ways to remove PFAS, they have no by-products, so nothing needs to be disposed of, and they can be used effectively in water treatment plants [16]. Many systems contain reverse osmosis membranes to treat their water, so this solution is effective, as governments can easily attach this to treat their water at a low cost. Granular activated carbon is effective in removing PFAS and can be reactivated once used. The combination of activated carbon and reverse osmosis membranes is the most effective and most common way water companies are removing PFAS from their waters [17]. However, the question is which one is viable; activated carbon is the cheaper alternative, but the question is whether it is viable. To test this, we plan to measure the influent and effluent water of the Palmyrita treatment plant and measure whether the levels of PFAS are high enough, where reverse osmosis is a better choice [18].

## Materials and methods

This study followed the US Environmental Protection Agency's Method 533 for the detection of PFAS in drinking water [19]. All testing was performed according to recommended guidelines to ensure accuracy and reproducibility.

Water samples were collected from three wells (Garner C, Twin Springs, and a private well), as well as influent and effluent points at the Palmyrita Treatment Plant in Riverside, CA. EPA-trained personnel flushed each water source before sampling to ensure stable readings and minimize contamination. Sterile 1-L polypropylene bottles were used to collect samples, which were stored at 4°C and transported to the testing facility within 48 hours.

Supplies needed

All materials and equipment used in this study are listed in Table 1, including consumables, analytical instruments, and reagents sourced from certified suppliers.

**Table 1:** List of supplies and equipment used in the experiment

Item	Quantity	Brand/Source	Location	Notes
Polypropylene bottles (1 L)	11	Sigma-Aldrich	California	
SPE cartridges (6 mL)	11	Sigma-Aldrich	California	
SPE Frits	33	Sigma-Aldrich	California	
Centrifuge tubes (15 mL)	—	Corning	California	Quantity not specified
Polypropylene bottles (20 mL)	—	Fisher Scientific	California	Quantity not specified
Analytical balance (Model: ME104E)	1	Mettler Toledo	California	
Eppendorf pipettes	—	Eppendorf	California	
HPLC vial (2 mL)	—	Fisher Scientific	California	Quantity not specified
Methanol	—	Fisher Scientific	California	
HPLC MS	1	Agilent	California	
Centrifuge (Model: 5424 R)	1	Eppendorf	California	
pH meter	1	Fisher Scientific	California	
Concentrator Plus	1	Eppendorf	California	
Vacuum extraction manifold	1	Phenomenex	California	
1290 Infinity II UHPLC	1	Agilent	California	
6470 Triple Quadrupole Mass Spectrometer	1	Agilent	California	
EPA Method 533 Isotopically Labeled PFAs (Lot PR-34325)	—	Cambridge Isotope Laboratories	Massachusetts	
MilliQ Water	—	Sigma-Aldrich	California	Ultra-pure, free of PFAs [20]

Sampling Procedure 

Water samples were collected from three wells (Garner C, Twin Springs, and a private well), as well as influent and effluent points at the Palmyrita Treatment Plant in Riverside, CA. EPA-trained personnel flushed each water source before sampling to ensure stable readings and minimize contamination. Sterile 1-L polypropylene bottles were used to collect samples, which were stored at 4°C and transported to the testing facility within 48 hours.

Analytical Methodology 

Samples were prepared using solid-phase extraction (SPE) with mixed-mode weak anion exchange (WAX) and cation exchange (WCX) cartridges. Analytes were eluted using methanol, spiked with isotopically labeled PFAS internal standards, and concentrated before analysis.

High-performance liquid chromatography coupled with tandem mass spectrometry (HPLC-MS/MS) was used to quantify PFAS levels. Calibration curves were established using PFAS standards across a range of 0.3-100 µM. The instrument used was the Agilent 1290 Infinity II UHPLC system coupled with a 6470 triple quadrupole mass spectrometer, operating in multiple reaction monitoring (MRM) mode.

**Figure 1:**
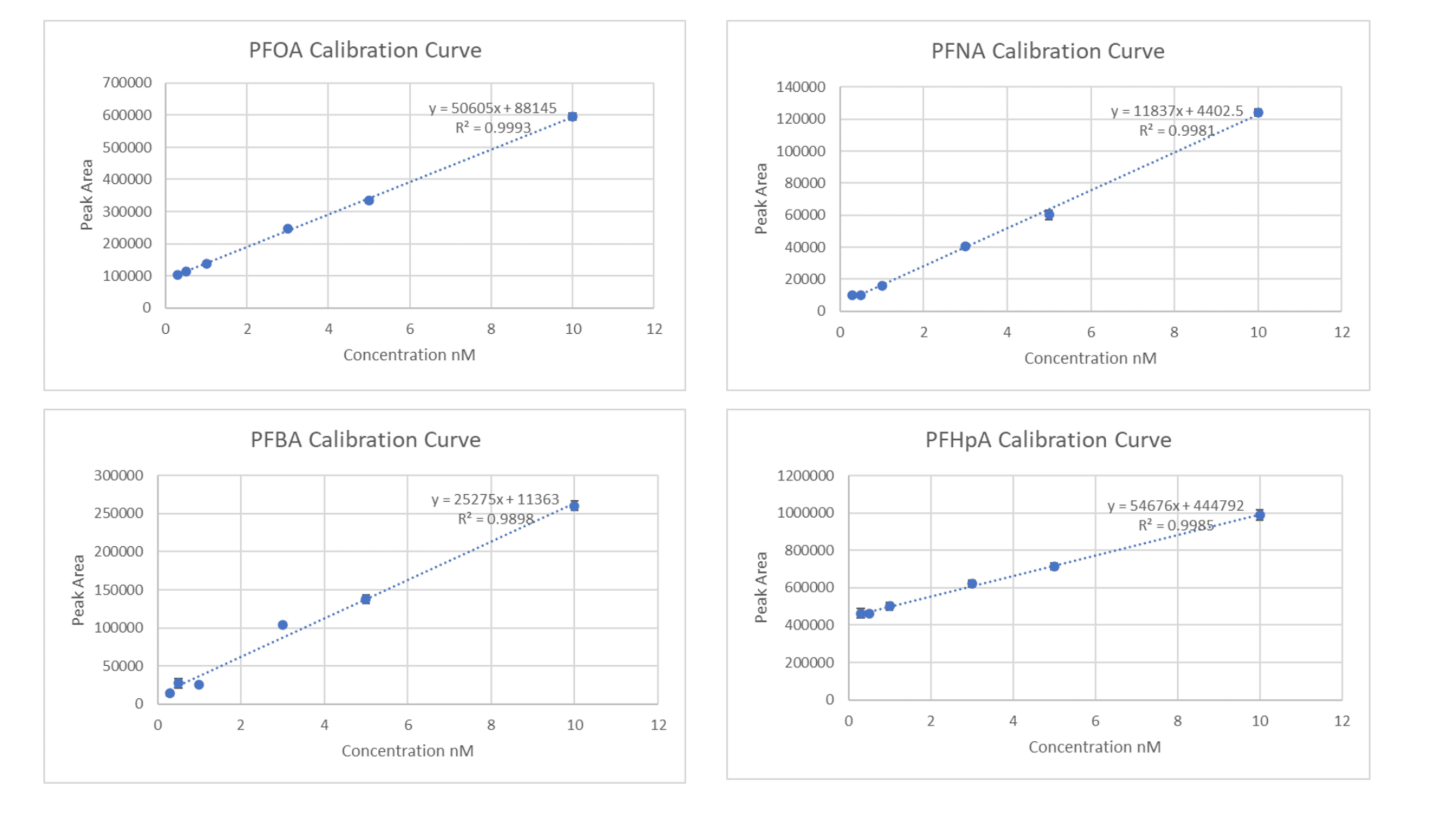
Calibration curves for PFOA, PFNA, PFBA, and PFHpA used in the experiment with error bars PFOA, perfluorooctanoic acid; PFNA, perfluorononanoic acid; PFBA, perfluorobutanoic acid; PFHpA, perfluoroheptanoic acid

**Figure 2:**
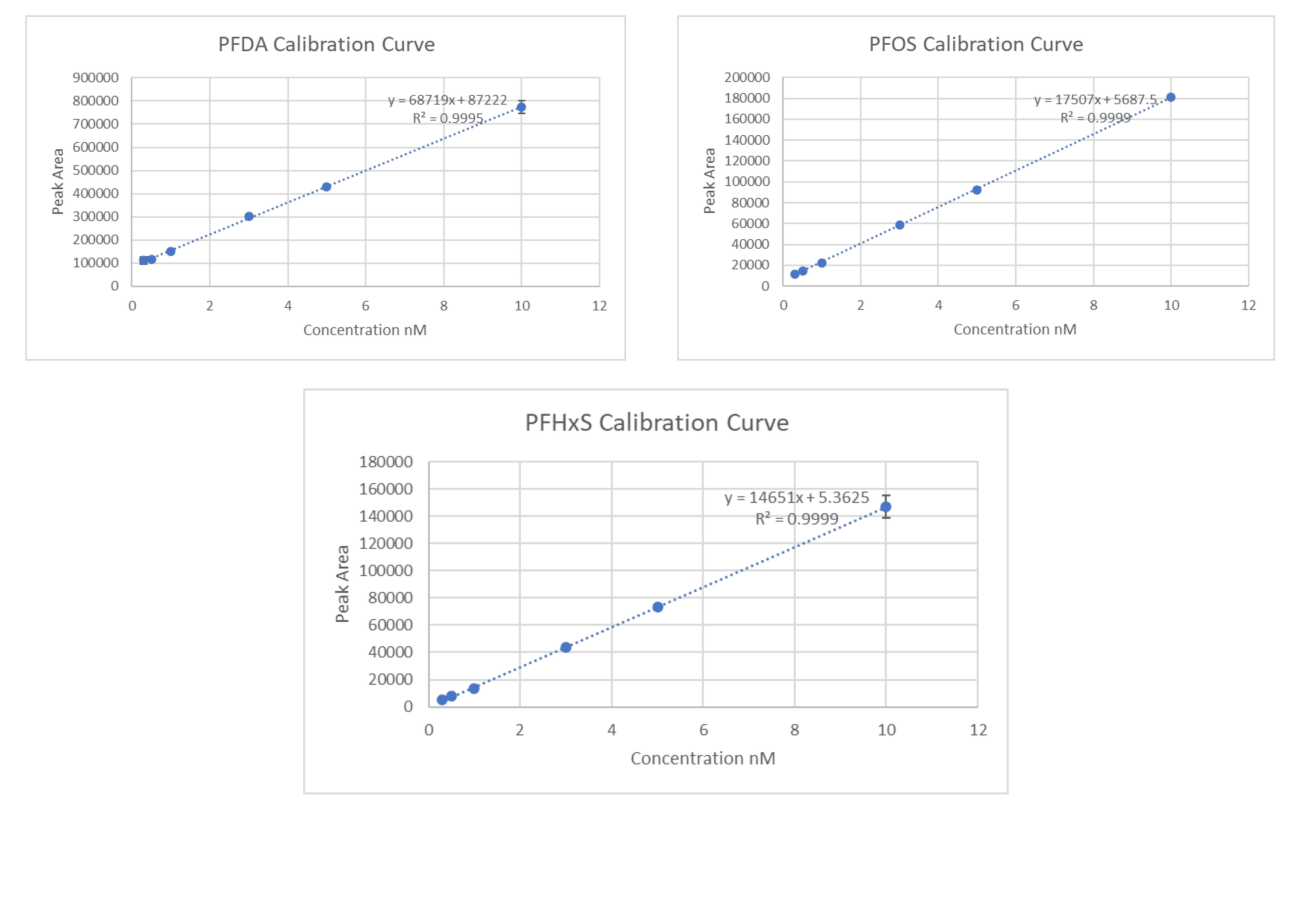
Calibration curves for PFDA, PFOS, and PFHxS used in the experiment with error bars PFDA, perfluorodecanoic acid; PFOS, perfluorooctane sulfonate; PFHxS, perfluorohexane sulfonate

To validate the calibration process and quantify PFAS concentrations, we created calibration graphs with the peak areas on the Y-axis and the concentrations on the X-axis. Using the measured peak areas from our samples, we applied the standard curve equation to determine PFAS concentrations. Results are shown in Figures 1-2.

Recovery efficiencies were monitored using labeled internal standards and blanks. The recoveries used in this experiment were 99.9%. Ideally, the recovery efficiencies should have been 100%, but the nature of working with small chemicals in this experiment lowered it to 99.9%. Results were quantified in ng/L. Data analysis and calibration were performed using Agilent MassHunter software.

Control samples using MilliQ water were processed alongside test samples to assess potential contamination during preparation and analysis. Duplicates were tested for each location to ensure reproducibility, and standard deviations were calculated to assess precision.

Analysis

We spiked a methanol tube with isotopic PFAS standards, which we could track the levels for and create a calibration curve. We created a calibration curve for each molecule we tested and then used the peak area for each molecule to get the respective concentrations of PFAs in our water. To ensure correct results, we made sure to create two methanol tubes, one for perfluorocarboxylic acids (PFCAs) and one for perfluorosulfonic acids (PFSAs), to compare more easily.

## Results

**Figure 3:**
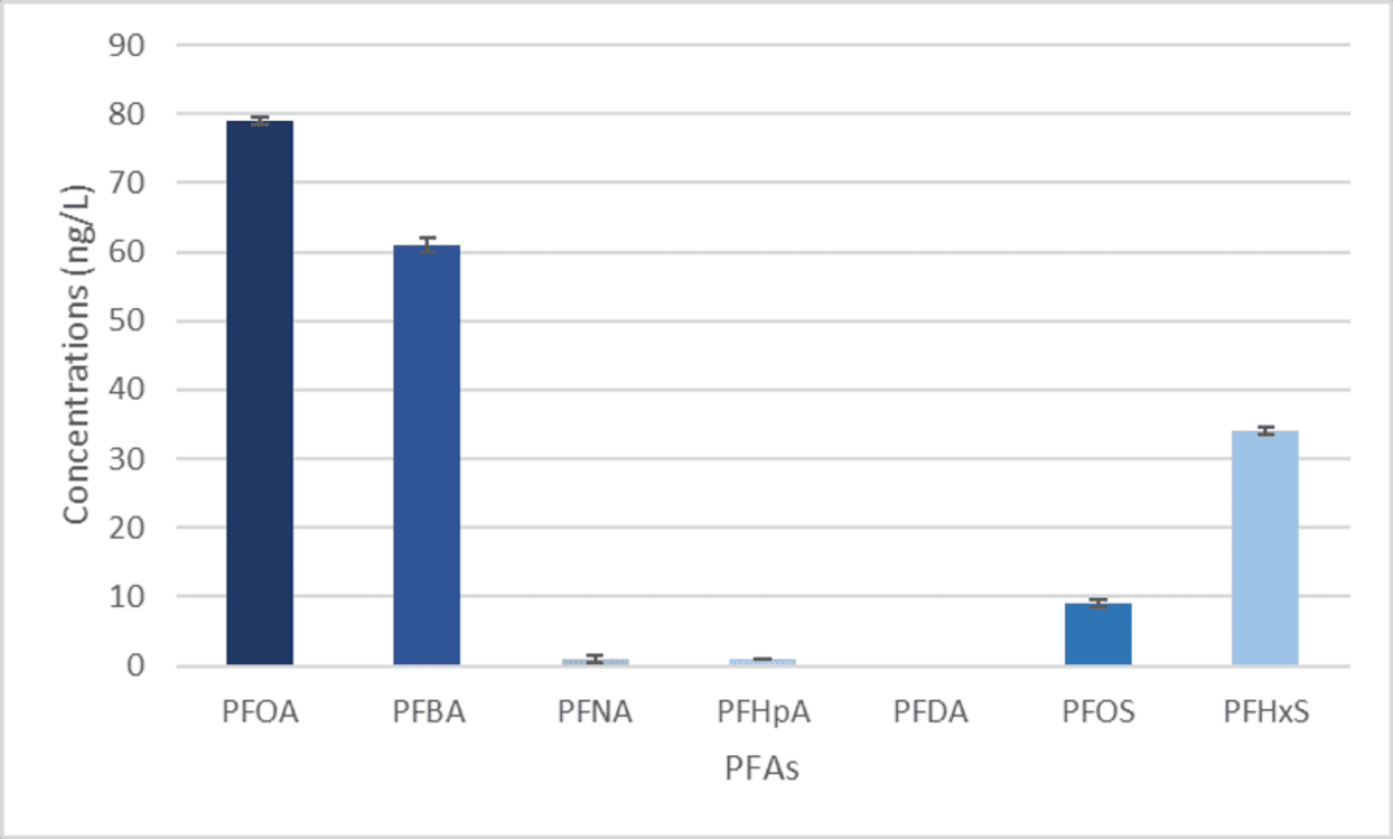
PFAS levels found in private wells PFOA, perfluorooctanoic acid; PFNA, perfluorononanoic acid; PFBA, perfluorobutanoic acid; PFHpA, perfluoroheptanoic acid, PFDA, perfluorodecanoic acid; PFOS, perfluorooctane sulfonate; PFHxS, perfluorohexane sulfonate

The reason for the high amounts of PFOS could be tied to the history of grain and orange farming in Woodcrest and Orangecrest due to the amount of pesticides used in the region [21,22]. Garbage on the streets is also a major factor in PFOA amounts because, during rainstorms, rain can pick up PFAS molecules and seep into our underground water over time. The dry climate in California also increases the risk of fires, and AFFF, a fire-fighting foam, is a major contributor to PFAS. Woodcrest has had many fires in the past, so this could be another big reason for the high PFOA amount [23,24]. This is supported by the PFAS concentrations shown in Figure 3.

**Figure 4:**
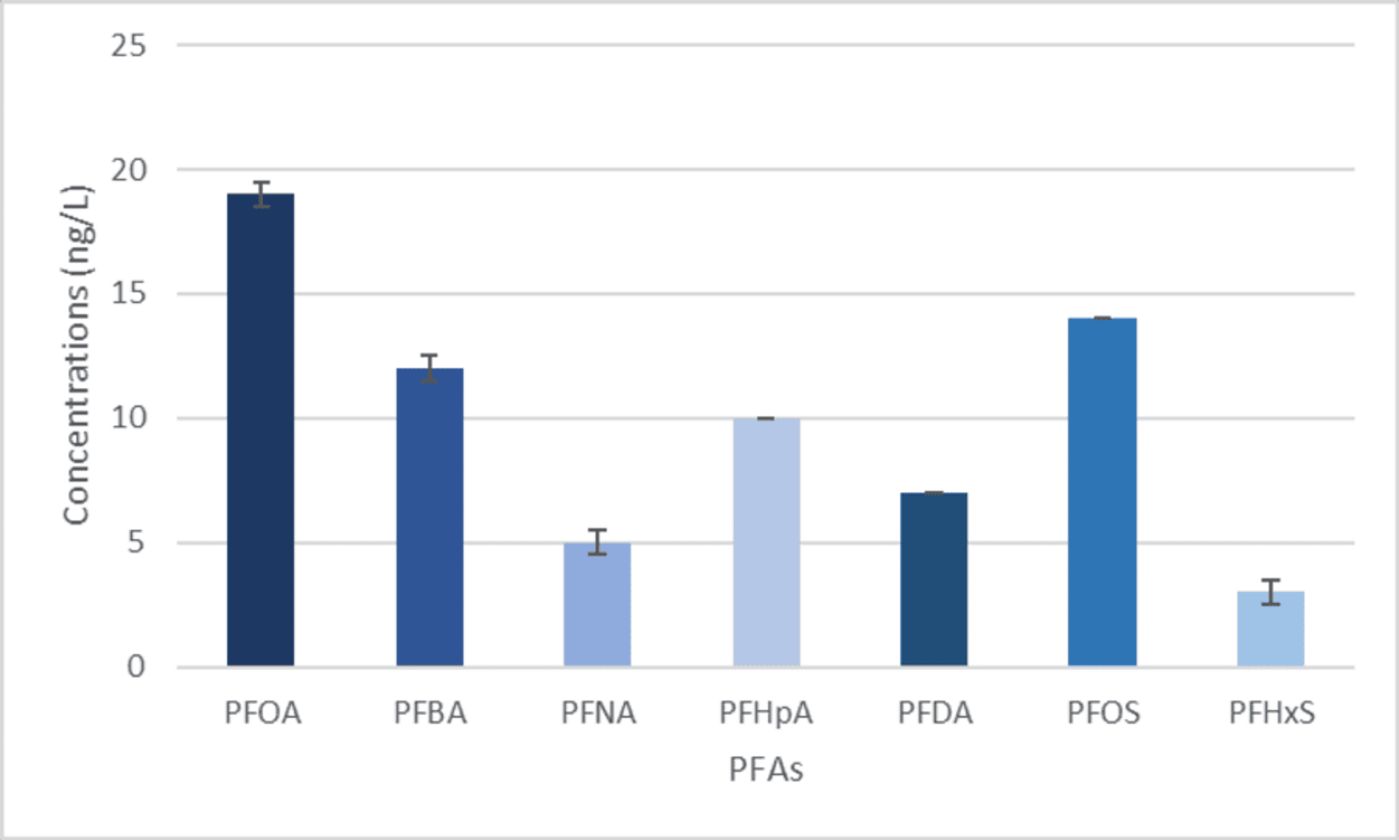
PFAS levels found in the Garner C well PFOA, perfluorooctanoic acid; PFNA, perfluorononanoic acid; PFBA, perfluorobutanoic acid; PFHpA, perfluoroheptanoic acid, PFDA, perfluorodecanoic acid; PFOS, perfluorooctane sulfonate; PFHxS, perfluorohexane sulfonate

**Figure 5:**
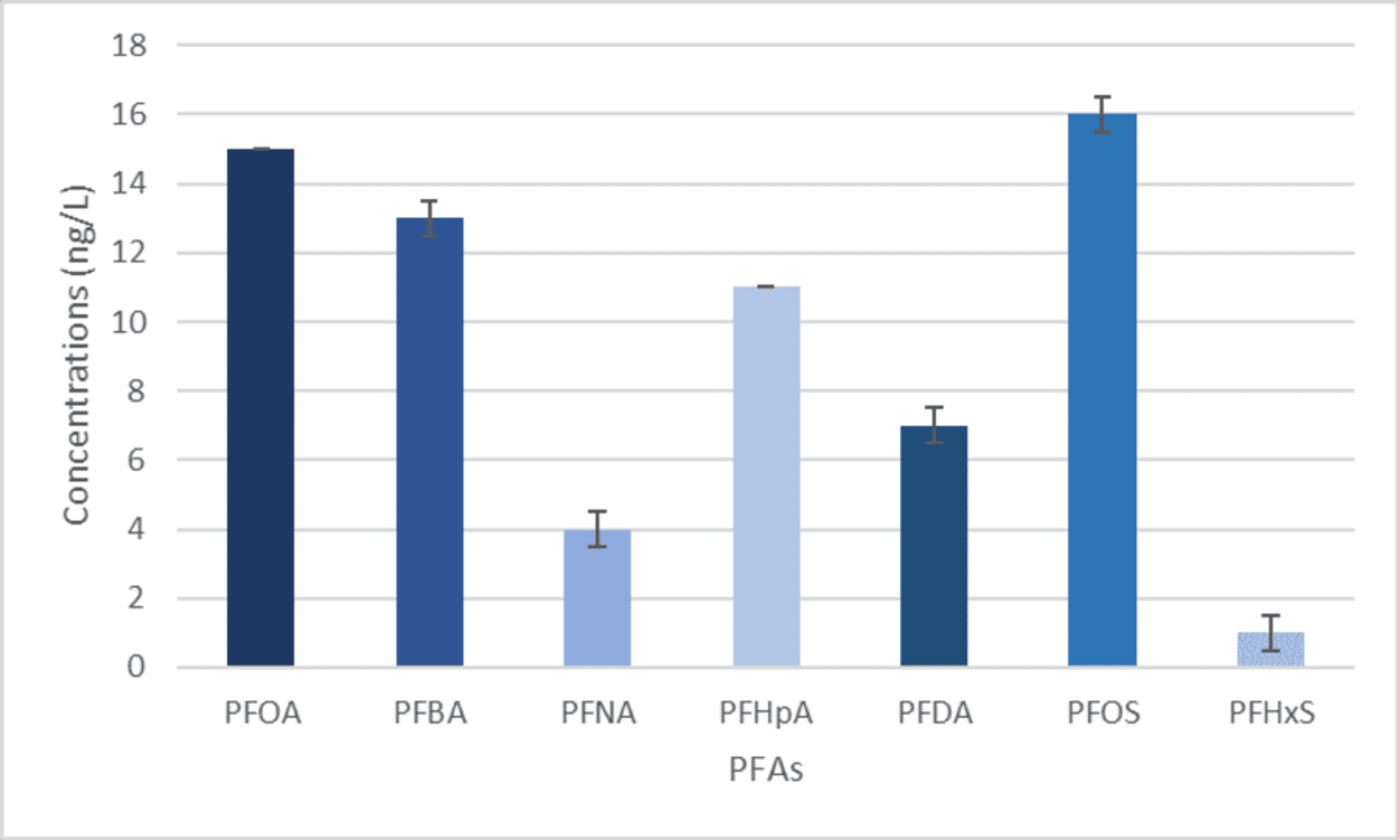
PFAS levels found in the Twin Springs well PFOA, perfluorooctanoic acid; PFNA, perfluorononanoic acid; PFBA, perfluorobutanoic acid; PFHpA, perfluoroheptanoic acid, PFDA, perfluorodecanoic acid; PFOS, perfluorooctane sulfonate; PFHxS, perfluorohexane sulfonate

Due to the Garner C well and the Twin Springs well being nearby, it justifies their similar PFAS amounts, as seen in Figures 4-5. Both wells had similar amounts of each chemical and similar numbers of each chemical. It is important to note that these samples were taken in a very urban and industrial-rich area, which would elevate the level of PFAs in the region [25]. Storm runoff and treated discharged wastewater go into the Santa Ana River, contaminating it with PFAS [26]. This water inevitably ends up in the underground water plume. Due to the proximity of the Santa Ana River has with the Garner C and Twin Springs Wells, it is an important contributor to contamination. Runoff from manufacturing industries and warehouses is also a probable reason for PFAS contamination. PFAS chemicals used during manufacturing and storage get gathered and moved by rain and eventually end up in stormwater drains that lead to the Santa Ana River [25]. The proximity to a landfill only further proves the high level and variety of PFAS. As stated previously, the rain picks up large amounts of PFAS as it moves along the ground. Because landfills have astronomically large amounts of trash that contain PFAS and other products with chemicals such as PFNA, it would make sense that landfills ultimately cause large amounts of contamination to the underground water plume [27].

Replicates, standard deviation, and error bars

For this experiment, each sample had duplicates. Where A and B correlated with one location, C and D correlated with one location, E and F correlated with one location, etc. The means of these numbers were plotted, with each bar indicating the result for a sample. There were no serious outliers in this experiment due to the precision of the equipment and diligence of the experimenters, so the mean was plotted instead of the median. The standard deviation was calculated and then used to create the error bars.

Influent and effluent water

**Figure 6:**
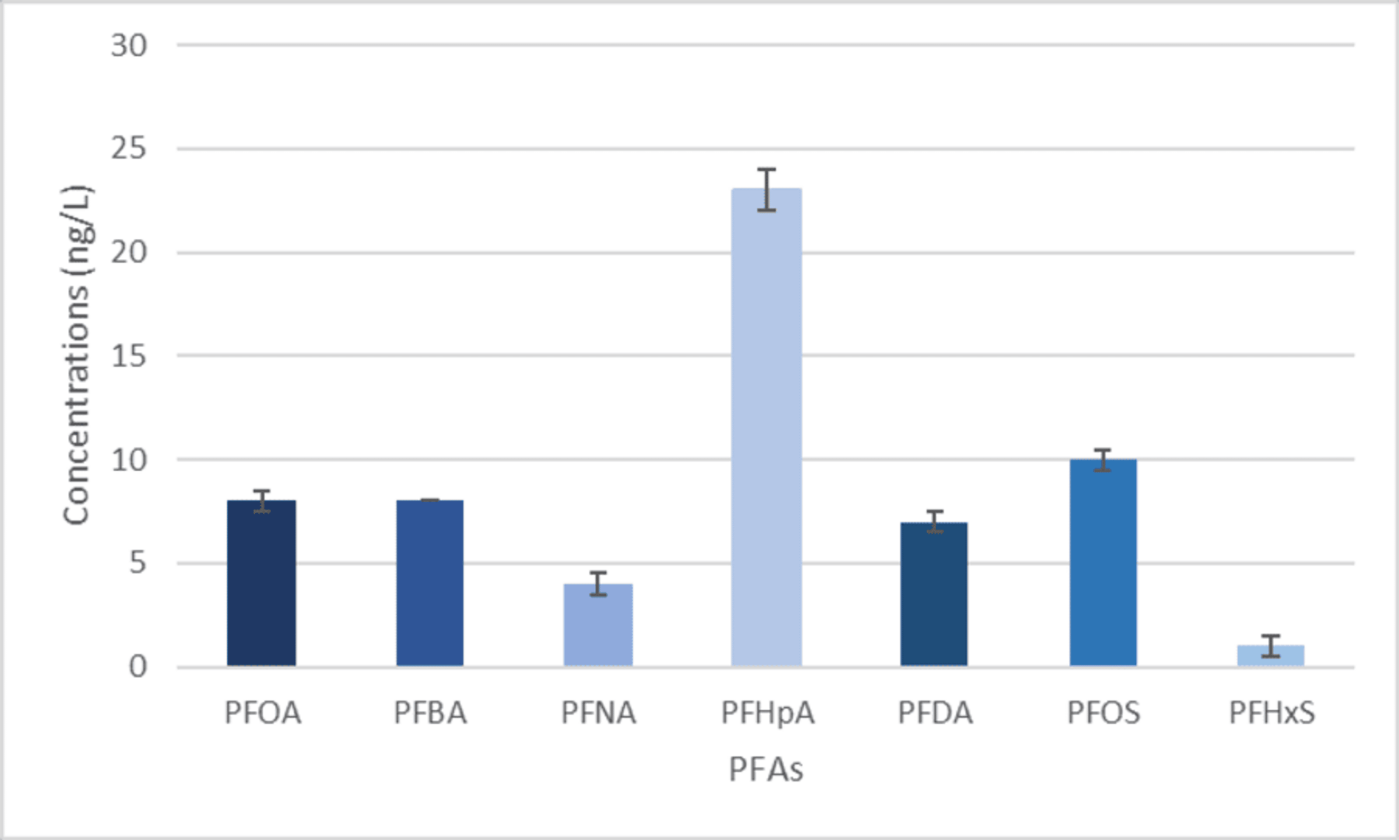
Original water PFAS concentration before treatment PFOA, perfluorooctanoic acid; PFNA, perfluorononanoic acid; PFBA, perfluorobutanoic acid; PFHpA, perfluoroheptanoic acid, PFDA, perfluorodecanoic acid; PFOS, perfluorooctane sulfonate; PFHxS, perfluorohexane sulfonate

**Figure 7:**
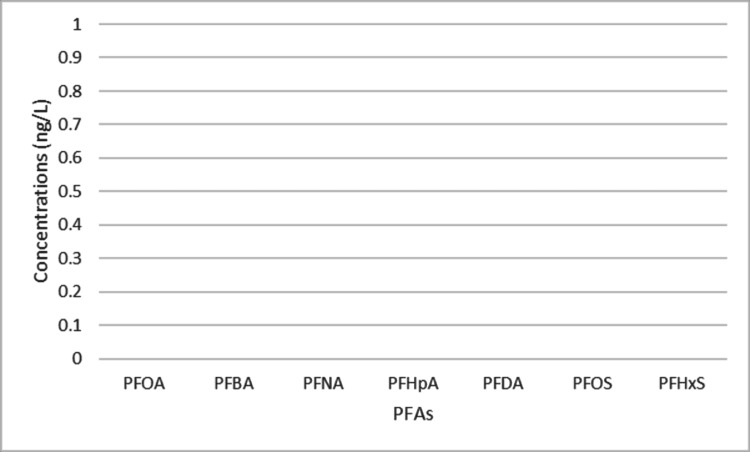
The removal of PFAS after going through the treatment plant PFOA, perfluorooctanoic acid; PFNA, perfluorononanoic acid; PFBA, perfluorobutanoic acid; PFHpA, perfluoroheptanoic acid, PFDA, perfluorodecanoic acid; PFOS, perfluorooctane sulfonate; PFHxS, perfluorohexane sulfonate

By testing the influent and effluent water for the Palmyrita Treatment Plant, we determined that Riverside’s usage of activated carbon to treat PFAS was effective. The influent water from the Palmyrita Treatment Plant had PFAS numbers of around 10 ng/L for each chemical (Figure 6); meanwhile, the effluent water had 0 ng/L of PFAs for each chemical (Figure 7). The Palmyrita Treatment Plant treats its water using activated carbon, so our project has concluded that activated carbon is effective in treating PFAs, as it removes all traces of PFAs. Although it is the cheapest way to clean PFAS in treatment plants, it was still incredibly effective in doing so. This method of cleaning PFAs in drinking water helps people, but it cannot be brought onto a larger scale to clean the environment.

Control

Our control sample shows an indication of 0 PFAs for both replicates. This proves that MilliQ water has 0 PFAs and is efficient in testing and cleaning in the discovery of future solutions for PFAs. Secondly, this 0 number indicates that there was no pollution from the methodology in the treatments. Thus, no PFAS was found in these samples.

## Discussion

This study highlights the presence and concentrations of PFAS in Riverside municipal and private wells, demonstrating the widespread contamination of groundwater by these persistent chemicals. Our findings confirm that PFAS levels in some sampled wells exceeded the EPA's recommended limit of 4 ppt for PFOA and PFOS, posing significant public health and ecological risks. These results align with previous research that underscores the pervasive nature of PFAS contamination in groundwater sources [28].

The data collected from the Palmyrita treatment plant showed variability in the removal efficiency of PFAS through the treatment process. While activated carbon and reverse osmosis membranes demonstrated potential in mitigating PFAS levels, challenges remain in ensuring consistent treatment efficacy across different well sources. The differences in PFAS concentrations between influent and effluent water emphasize the need for more robust and scalable treatment technologies to address this issue comprehensively. Previous studies have showcased the importance of protecting PFAs in groundwater sources, rather than simply treating them for human drinking water. Consequently, these findings suggest that, while Riverside's current water treatment strategies are partially effective, further optimization of its groundwater sites may be required to meet public health standards sustainably [28].

Limitations

While our study provides valuable insights into PFAS contamination in Riverside, it has certain limitations. First, the sample size was relatively small, with only a limited number of wells and treatment plants analyzed. A larger dataset encompassing more wells across different geographical areas would provide a more comprehensive understanding of regional PFAS contamination. Second, the study focused primarily on PFOA and PFOS, though other PFAS compounds may also contribute to contamination and health risks. Future studies should expand the scope to include a broader range of PFAS chemicals. Third, the reliance on laboratory-based testing methods, while precise, may not fully capture the variability in PFAS levels due to seasonal or usage-related factors. Finally, this study did not include an economic analysis of treatment methods, which is critical for assessing the feasibility of large-scale implementation. Addressing these limitations in future research could provide a more holistic view of PFAS contamination and mitigation strategies.

Although only 15% of private well owners use private well water as a primary source of drinking water in America, the majority of private well owners use private well water as a primary means of agriculture [29]. This number may seem low, but the 13 million Americans who rely on well water are at risk of serious bioaccumulation of PFAs in their body [29]. Furthermore, the vast majority of private well owners use their well water as a means of agriculture, but just because this water is being used for agriculture does not mean it cannot harm humans. Studies have proven that PFAs bioaccumulate within food and then travel up the food chain, to eventually reach humans. Due to the biomagnification of PFAs, humans are at risk of taking large amounts of PFAs when eating meat, which further amplifies the effects of PFAs within the human body [5]. In fact, the owner of the private well in our experiment removed thousands of her orange trees after learning of the levels of PFAs in her well water, due to this water being used to irrigate her crops.

Call for action

Our study also illuminates the broader implications of PFAS contamination, including its potential impact on human health and the environment. PFAS exposure is associated with a range of health issues, such as immune system dysfunction, cancer risks, and developmental effects in children [30]. The persistence of these chemicals in both the environment and the human body underscores the urgent need for proactive measures to minimize exposure and contamination. Although companies such as Riverside have till 2029 to fix their water, the need to fix it by 2029 and the human impacts it will cause in the meantime sparks concern. Essentially, there is no safe level for PFAs in drinking water, forcing the EPA to set the limit of clean drinking water to be 4 ppt, a number that represents the lowest level that current technology can reliably detect PFAs [31,32]. This makes it evident that companies such as Riverside remove their groundwater of PFAs to prevent further bioaccumulation and biomagnification from happening across species. Companies can do this through up-and-coming technologies or simple bioremediation, where microbes are used to treat a body of water [33].

## Conclusions

This study underscores the urgent need to address PFAS contamination in Riverside’s water supply. While current treatment methods, such as activated carbon and reverse osmosis, show promise in reducing PFAS levels, they require further optimization to achieve consistent and comprehensive removal. The widespread nature of PFAS contamination highlights the importance of implementing stringent regulations and exploring innovative treatment solutions to safeguard public health and environmental well-being. Future research should focus on expanding sampling efforts, analyzing additional PFAS compounds, and assessing the economic viability of treatment methods to inform effective policy and practice.
